# Trismus

**Published:** 1828-12

**Authors:** 


### TRISMUS.

### *Case of Trismus, conjoined with Paralysis of the Face, at the* MIDDLESEX HOSPITAL.

THOMAS JONES, æt. twenty-nine, a groom, was admitted under Mr. BELL's care, October 11th: he complained of a painful stiffness in his jaws, and the muscles of one side of his face were paralysed. He stated that, on the last day of September, while dressing his horse, it struck him with the fore foot upon the right side of his head, and knocked him down. He remained insensible for some time. When he returned to consciousness, he felt weak, and a little sick. There was a wound, as if made by the heel of the shoe, just over the external angular process of the frontal bone. Nothing, however, was done for him, and he lived as usual. On the fourth day after the accident, he first perceived that his face was twisted to one side; he then had also some difficulty in speaking and swallowing. It was not till the 6th October that he consulted a medical man, who recommended him to come to the hospital.

The face is twisted to the left side, as in the cases of partial paralysis from injury to the portio dura; and this distortion of the face is most observable when he speaks. Upon being asked to close his eyes, the left is shut, but the eyelids of the right side are very imperfectly closed, and in the attempt the cornea is turned up. *The feeling on the right side of the face is as perfect as on the left.* It cannot be perceived how far the motion of the tongue is impeded, as he cannot open his mouth freely: he is apt to bite both his tongue and cheek while eating. The wound on the side of the orbit resembles a mere scratch, nearly healed. There was no bleeding from the ear after the accident, and he hears perfectly with both ears. There is a fulness and rigidity about the masseter muscle on the right side, and Mr. Bell thinks there is a preternatural swelling before the right ear.

**Hirudines xij. ante aurem.—Pil. Colocynth. cum Calom gr. x. statim, et mane haustus purgans.—Lotio Plumbi Acet. cum Opio ad partem dolentem.**

11th October.—The house surgeon was called in the morning to this patient. He found him struggling like one who is suffocated. He seemed to labour from a difficulty of expectoration; his jaws were firmly clenched; his face was livid; the muscles on the right side were relaxed and drawn to the left side; those of the neck were rigid, and in strong action. It required the power of two men to restrain him in bed. Two drachms of the tincture of opium were administered in small quantities between his teeth, after which the fit subsided. Today his jaws are more firmly closed. He complains of a pain at the back of his neck, as if something were dragging or pinching him there. His bowels have been opened. Pulse 110, and firm.

**Cucurb. cruent. occipiti.—Hydrarg. Submur. gr. x.—Tinctura Opii ʒss. tertiis horis.**

12th October.—The patient today was visited by Drs. LATHAM, WATSON, and HAWKINS. The teeth are more closed. The suffering of which he complains most is from the phlegm in his throat, which makes him cough, and he throws out his saliva as in hydrophobia. During the paroxysms he starts up in bed; and we find him now sitting on the side of it, unwilling to lie down, as he is afraid of a recurrence of the fits.

Capiat Hydrarg. Submur. gr. x.—Enema Opii.—Cucurb. cruent. nuchæ ad ℥x.—Descendat in baln. calid.—Cataplasma cum Lotione Plumb. Acet. cum Opio ad vulnus.—℞. Extract. Tabaci; Unguent. Hydrarg. part. æqual. fiat unguentum. This ointment to be rubbed upon the neck and jaws.

13th.—Yesterday he was put into the warm bath, which was followed by a copious perspiration, and he expressed himself relieved by it. The fits attacked him four or five times during the day, and they continued about five minutes each time. He was unable to speak during them. His head was thrown back and his chin tilted up, but not so much as to be called opisthotonos. He has never complained of spasms in his epigastrium. He possessed a perfect command over his arms, legs, and head; but he had convulsive twitchings as he lay in bed. About seven in the evening his jaw began to be relaxed, but this was accompanied with evident symptoms of approaching dissolution. He sunk gradually, after having had several severe fits, and died this morning at ten o'clock.

*Dissection*, twenty-four hours after death.—When the brain was examined, the tunica arachnoidea was found slightly opaque, and the veins were more turgid with blood than natural. There was also some serum in the ventricles; but in other respects, on a close examination of this organ, and of the nerves coming from it, the appearances were perfectly healthy. The roots of the fifth pair of nerves, and the course of the portio dura through the temporal bone on the right side, were carefully examined, without detecting any alteration from their natural structure. The spinal marrow seemed healthy. The nerves of the sympathetic system (in the abdomen and the chest) were examined, without discovering any thing preternatural. The viscera, both of the thorax and abdomen, were in a healthy state, and the lungs were not more gorged with blood than is common. The glandulæ truncatæ at the root of the tongue were enlarged; but there was no redness marking inflammation either in the fauces, or larynx, or œsophagus.

Mr. Bell, in his observations on this case, first remarked its resemblance to some cases of partial paralysis of the face, in which he had been consulted during the present season. He admitted that the incapacity of closing the eye, and the total loss of motion of the lips and cheek on one side, deceived him when he first saw this patient in the waiting room. The anomaly of the case was, that on the side where the hurt had been received, the exterior muscles of the face, all those influenced by the portio dura, were in a state of paralysis; whilst the muscles of the jaws, supplied by the fifth pair, were in a state of tetanic spasm. Mr. Bell related a case of paralysis of the muscles of the face on one side, produced by a blow upon the head; but he added that, in the present case, on looking retrospectively, there was no reason to suppose the symptoms referrible to an injury of the brain, much less to an injury of the nerve passing through the bone: it was, he conceived, a case of trismus, arising from the slight bruise of the integuments of the temple operating upon a constitution morbidly predisposed.

